# Comparative Evaluation of Micro-tensile Bond Strength in Non-carious Lesions After Different Pre-surface Treatments

**DOI:** 10.7759/cureus.69963

**Published:** 2024-09-22

**Authors:** Jayaraj Mohan Dhas, Sihivahanan Dhanasekaran, Vijay Venkatesh

**Affiliations:** 1 Conservative Dentistry and Endodontics, SRM (Sri Ramaswamy Memorial) Kattankulathur Dental College, Chennai, IND

**Keywords:** adhesive, bond strength, citric acid, etchant, microtensile strength, noncarious lesion, phosphoric acid

## Abstract

Background: Non-carious cervical lesions (NCCLs) present themselves as smooth saucer lesions with variable height and depth. Treatment of choice for these lesions should be aimed at conservative management by restoring the teeth. The present study aims to evaluate the micro-tensile bond strength in NCCLs after various surface pre-treatments.

Materials and methods: Sixty-six human permanent maxillary first premolars were subjected to artificial wedge-shaped lesions on the buccal aspect of the cervical regions of all the teeth. Samples were divided into two groups (n = 33 per group). Group 1 received a 37% orthophosphoric acid treatment for 30 seconds and a 17% ethylene diamine tetraacetic acid (EDTA) application for 10 seconds. Group 2 received a 10% citric acid treatment for 30 seconds, followed by a 17% EDTA application for 10 seconds. These groups were further subdivided according to the bonding agent applied as subgroup A: 3M™ Single Bond™; Subgroup B: Prime & Bond Universal group; and Subgroup C: Tetric N-Bond Universal. Samples were subjected to incremental restoration and then segmented to fit into Geraldelli jig with cyanoacrylate adhesive. A universal testing machine was used to assess the micro-tensile bond strength and the data obtained were subjected to statistical analysis.

Results: In this study, statistically significant differences in micro-tensile strength were observed between the pre-treatment groups and the effect of the bonding agent. Consequently, the null hypothesis was rejected, and the research hypothesis was accepted.

The micro-tensile strength for both groups was compared with various pre-conditioning methods. It was noted that the micro-tensile strength was maximum when Tetric N Bond was used along with 37% orthophosphoric acid followed by Prime Bond with highly statistically significant values (p = 0.000). Contrary to this, in the 10% citric acid group, the micro-tensile strength was equally comparable in the 3M ESPE and Tetric N bond groups compared to the Prime Bond group, with highly statistically significant differences (p = 0.000).

Conclusion: Surface pre-treatment in NCCLs improves the bonding strength of the restoration used. Analysis using scanning electron microscopy and long-term assessment of the failure of restoration should be carried out in future studies.

## Introduction

A new clinical entity that occurs on the cervical aspect of root surfaces with no evidence of carious lesions is known as “non-carious cervical lesions (NCCLs)” [1]. The lesions are characterised by a loss of mineralised tissue at the tooth surface along the margin of the gingiva. This demineralisation extends from the cementoenamel junction onto the root surface. These lesions can vary in depth and shape, presenting as shallow, concave, wedge-shaped, notched, and irregular in appearance [2]. They may be associated with dentinal sensitivity [3], although some lesions exhibit limited or no sensitivity [4,5].

Since these lesions are non-carious, confusion exists among practitioners whether to intervene or to wait till what degree of wear before considering intervention. Published recommendations state that “preventive interventions may be preferable to extensive restorative care and the latter to be delayed as long as possible” [6,7]. It also suggests that the “decision to monitor NCCLs rather than intervening in them should be based on the progression of the lesion and how these lesions compromise tooth vitality, function, and aesthetics” [8].

NCCLs can be restored with the help of various restorative materials with composite resins indicated commonly for their aesthetic and clinical properties [8]. These materials effectively protected the affected tooth structure from further deterioration and demonstrably reduced dentinal hypersensitivity [9,10]. Despite its advantages, restoration failure due to debonding of the resin material from the sclerotic dentin and inadequate establishment of a hybrid layer has been reported in studies with a range of restoration failures from 0% to 50% [11,12].

To overcome these problems and improve the adhesion of composite resins, mechanical removal of surface dentin can be done; it increases the hybrid layer thickness thereby increasing the retention rates of composite resins in NCCLs [13,14].

Hence, to improve the bond strength, various pre-treatment methods on dentin have been proposed, such as acid etching or air abrasion techniques. The acid etching procedure is used primarily for smear layer removal and demineralisation as well. This procedure influences the adhesive bonding as the exposed collagen fibres after etching are vulnerable to hydrolytic and enzymatic degradation [15-17]. Hence, the aim of the research is to investigate the micro-tensile bond strength in non-carious lesions after various surface treatments. The present study investigates the micro-tensile bond strength in non-carious lesions after various surface treatments with a null hypothesis of no difference in the micro-tensile bond strength in the pre-conditioning group subjected to different bonding agents. The alternative hypothesis states that there is a statistically significant difference between the pre-conditioning groups and the bonding agents used.

## Materials and methods

Tooth selection and preparation

**Figure 1:**
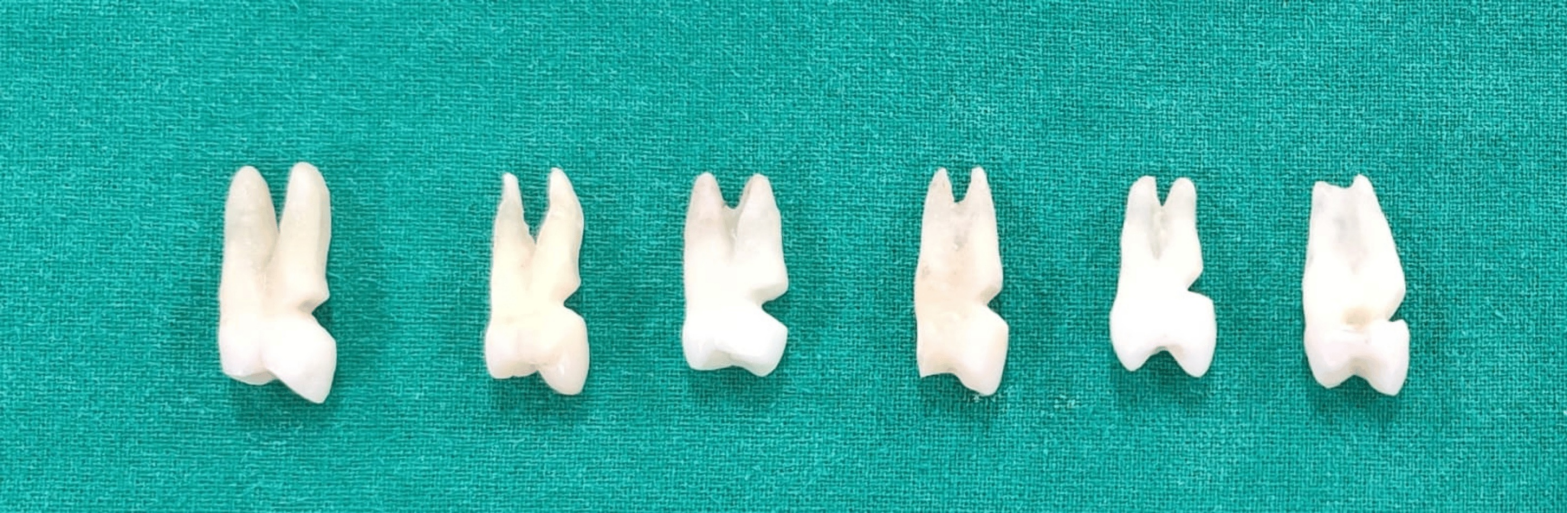
Prepared cervical lesions in the extracted tooth using fine-grit, cone-shaped diamond bur

This study was carried out after obtaining ethical committee clearance from the institutional review board (SRMIEC-ST1023-1387). Sixty-six human permanent maxillary first premolars extracted due to periodontal disease or for orthodontic purposes after receiving patients' consent were included in this study. The study was carried out in the SRM Medical Science and Research Institute from December 2023 to February 2024. All extracted teeth were meticulously cleaned using an ultrasonic scaler to remove any visible plaque or calculus on the tooth surface. Following this, standardised wedge-shaped lesions of well-defined dimensions were created on the buccal aspect of the cervical region of all the teeth using fine-grit, cone-shaped diamond bur and then stored in a container at 40 degree Celsius for a month before being tested (Figure 1).

The G*Power software (3.1.9.2 version; Heinrich Heine University, Dusseldorf, Germany) was used to estimate the sample size in the current study. Considering a priori power analysis, F tests - analysis of variance (ANOVA): fixed effects, omnibus, one-way and the input parameters α error as 0.05 at 95% CI, β error as 0.05, power of the test (1-β error) as 95%, number of groups as 3, and effect size (Cohen’s f statistic) as 0.5012, a total sample size of 66 (n) was estimated for the current study with an output actual power of 95.34%. 

Pre-treatment methods and classification of groups

To assess the effect of the pre-conditioning method, samples were divided into two groups (n = 33 per group). Group 1: pre-conditioning with 37% orthophosphoric acid for 30 seconds, followed by 17% ethylene diamine tetraacetic acid (EDTA) for 10 seconds. Group 2: pre-conditioning with 10% citric acid for 30 seconds, followed by 17% EDTA for 10 seconds. These samples were again subdivided into three groups (n = 11) according to the adhesive approach as subgroup A: 3M™ Single Bond; subgroup B: Prime & Bond Universal (Dentsply); and subgroup C: Tetric N-Bond Universal (Ivoclar).

Restoration of samples

Following the pre-treatment of the samples, adhesives were applied over the defect according to the manufacturer’s instructions. Air drying of the tooth surface was done for 5 seconds and a light-emitting diode (LED) light operated at an intensity of 1600 mW/cm^2^ was used to cure the composite (3M™ Filtek™ Z350 XT Universal Restorative composite (3M ESPE, USA)) for 10 seconds.

Assessment of micro-tensile bond strength

**Figure 2:**
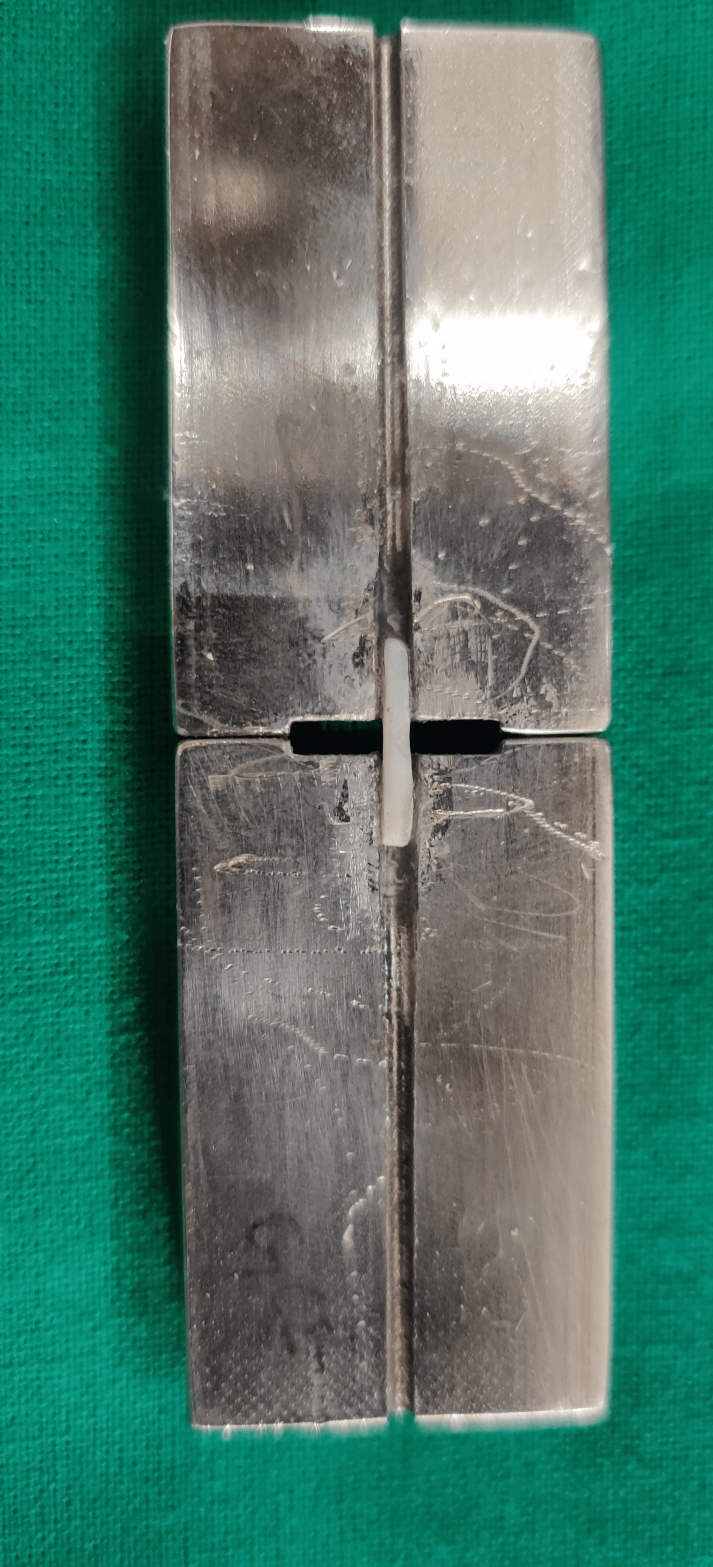
Geraldelli jig with samples to be tested

The tooth was sectioned into a thickness of 1 mm using a diamond disc under water coolant. Segmented specimens were then attached to a specifically designed and modified Geraldelli jig with the help of cyanoacrylate adhesive (Figure 2).

**Figure 3:**
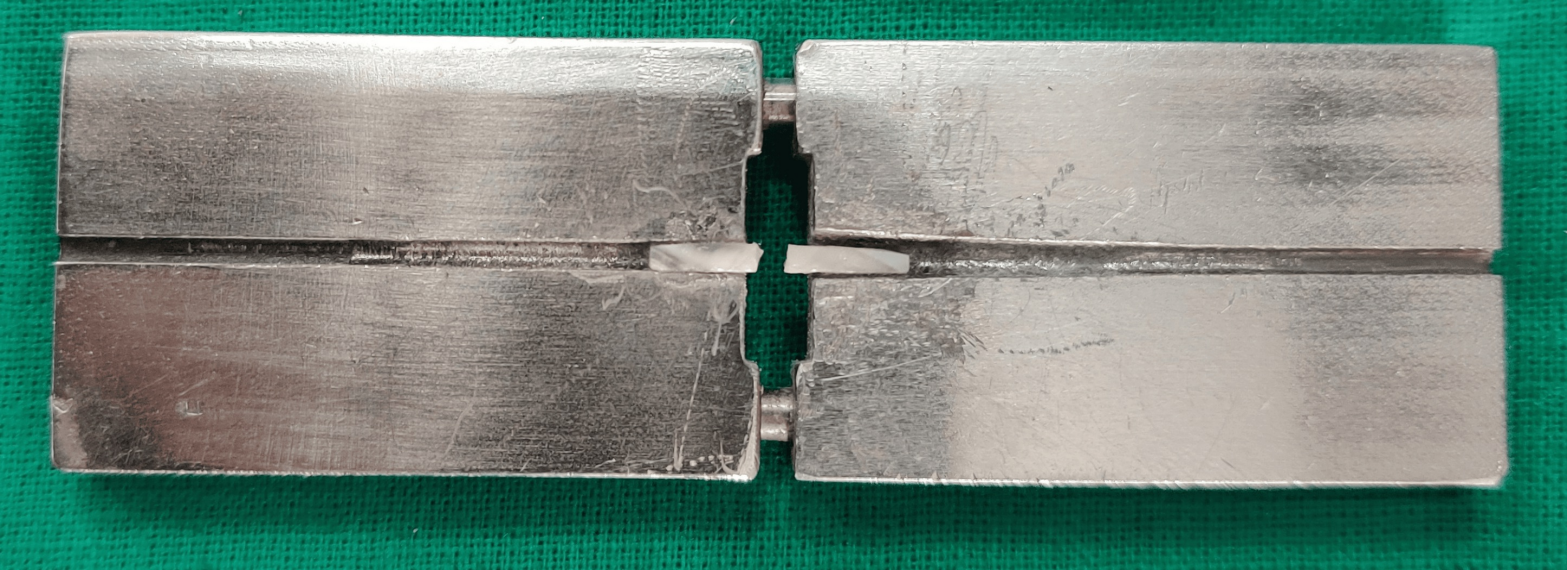
Fractured specimen after testing

The micro-tensile bond strength was evaluated using a universal testing machine (ElectroPuls E 3000; Instron, Norwood, MA). Glued specimens were pulled in tension at a cross-head speed of 0.5 mm/min using a micro-tensile tester (Isomet, Manassas, VA) until failure (Figure 3). The tensile strength values were recorded with Nexygen-MT (Lloyd Instruments, Steyning Way, Bognor Regis) and statistically analysed.

Statistical analysis

The normality of the data distribution was assessed using the Kolmogorov-Smirnov test (SPSS 20.0 software; IBM Corp, Armonk, NY). Descriptive statistics were calculated to characterise micro-tensile bond strength values, including means, standard deviation, and 95% confidence intervals. Intergroup comparisons among surface pre-treatment groups were performed using one-way ANOVA followed by Tukey’s honest significant differences (HSD) post hoc test for multiple pairwise comparisons. The level of significance was set at p ≤0.05.

## Results

The micro-tensile strength between etchants was compared while using three pre-conditioning methods. It was noted that the mean micro-tensile strength was maximum when Tetric N Bond was used along with 37% orthophosphoric acid (59.67 ± 3.01) and also with 10% citric acid (48.17 ± 2.67), whereas Prime Bond showed higher mean micro-tensile strength when used with 37% orthophosphoric acid (47.93 ± 5.60) and least with 10% citric acid. A higher mean micro-tensile strength was observed when 3M ESPE was used with 10% citric acid and least with 37% orthophosphoric acid. The differences between the mean micro-tensile strength while using pre-conditioning methods along with two different etchants were highly statistically significant (p = 0.000). The mean and standard deviation values of micro-tensile strength and the lower and upper confidence interval for mean values at 95% are described in Table 1.

**Table 1:** Intergroup comparisons of micro-tensile bond strength between different etching and pre-conditioned groups Group 1 - 37% orthophosphoric acid. Group 2 - 10% citric acid. *Statistically significant (p ≤ 0.05) for F-value.

Group	Subgroup	n	Mean ± SD	95% confidence interval for mean		F-value (p-value)
				Lower	Upper	
Group 1	Subgroup A: 3M ESPE	11	33.46 ± 3.07	31.39	35.53	85.627 (0.000)*
	Subgroup B: Prime Bond	11	47.93 ± 5.60	44.17	51.70	
	Subgroup C: Tetric N Bond	11	59.67 ± 3.01	57.64	61.69	
Group 2	Subgroup A: 3M ESPE	11	45.15 ± 3.09	43.07	47.23	
	Subgroup B: Prime Bond	11	35.91 ± 1.61	34.83	37.00	
	Subgroup C: Tetric N Bond	11	48.17 ± 2.67	46.37	49.96	
Total		66	45.05 ± 9.31	42.76	47.34	

The post hoc Tukey HSD test was performed following a significant one-way ANOVA test. The post hoc analysis revealed multiple pairwise intergroup mean differences between the etchant and pre-conditioning groups. Highly statistically significant mean differences in the micro-tensile strength were noted among certain included groups (p = 0.000) as described in Table 2. The highest mean difference was observed between 3M ESPE and Tetric N Bond when used with 37% orthophosphoric acid (26.20). The least mean difference was between 3M ESPE and Prime Bond when used with 10% citric acid (9.23).

**Table 2:** Intergroup comparisons of micro-tensile bond strength between different etching and pre-conditioned groups Group 1 - 37% orthophosphoric acid. Group 2 - 10% citric acid. *Mean difference is significant at the 0.05 level.

Main groups (I)	Comparison groups (J)	I-value	J-value	Mean difference (I-J)	p-Value	95% Confidence interval	
						Lower	Upper
Etching 1 + 3M ESPE	Etching 1 + Prime Bond	33.46	47.93	-14.47273^*^	0.000	-18.7388	-10.2067
	Etching 1 + Tetric N Bond		59.67	-26.20909^*^	0.000	-30.4751	-21.9431
	Etching 2 + 3M ESPE		45.15	-11.69091^*^	0.000	-15.9569	-7.4249
	Etching 2 + Prime Bond		35.91	-2.45455	0.541	-6.7206	1.8115
	Etching 2 + Tetric N Bond		48.17	-14.70909^*^	0.000	-18.9751	-10.4431
Etching 1 + Prime Bond	Etching 1 + Tetric N Bond	47.93	59.67	-11.73636^*^	0.000	-16.0024	-7.4703
	Etching 2 + 3M ESPE		45.15	2.78182	0.401	-1.4842	7.0478
	Etching 2 + Prime Bond		35.91	12.01818^*^	0.000	7.7522	16.2842
	Etching 2 + Tetric N Bond		48.17	-0.23636	1.000	-4.5024	4.0297
Etching 1 + Tetric N Bond	Etching 2 + 3M ESPE	59.67	45.15	14.51818^*^	0.000	10.2522	18.7842
	Etching 2 + Prime Bond		35.91	23.75455^*^	0.000	19.4885	28.0206
	Etching 2 + Tetric N Bond		48.17	11.50000^*^	0.000	7.2340	15.7660
Etching 2 + 3M ESPE	Etching 2 + Prime Bond	45.15	35.91	9.23636^*^	0.000	4.9703	13.5024
	Etching 2 + Tetric N Bond		48.17	-3.01818	0.310	-7.2842	1.2478
Etching 2 + Prime Bond	Etching 2 + Tetric N Bond	35.91	48.17	-12.25455^*^	0.000	-16.5206	-7.9885

**Figure 4:**
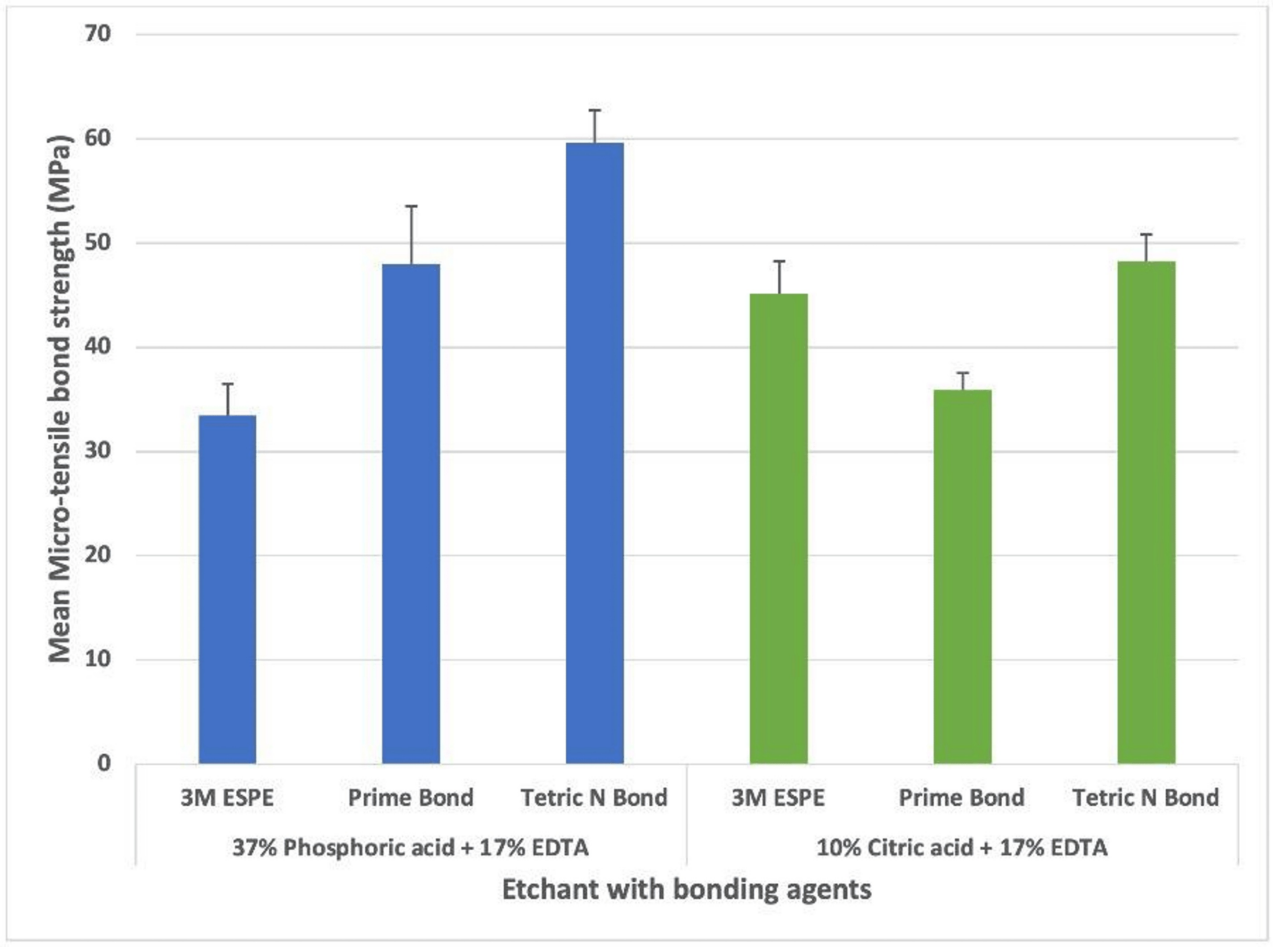
Micro-tensile strength between various pre-conditioning agents and the etchants used measured in terms of MPa EDTA, ethylene diamine tetraacetic acid; MPa, megaPascal.

The micro-tensile strength between various pre-conditioning agents and the etchants used is depicted in Figure 4. The 37% orthophosphoric acid group was observed to have better micro-tensile strength when the Tetric N bond and Prime Bond agents were used. Contrary to this, a higher micro-tensile strength was noted when the Tetric N bond and 3M ESPE agents were used along with 10% citric acid as an etchant.

In this study, statistically significant differences in micro-tensile strength were observed between the pre-treatment groups and the effect of the bonding agent. Consequently, the null hypothesis was rejected and the research hypothesis was accepted.

## Discussion

NCCL causes wear of the enamel of teeth, subsequently exposing the dentin underneath it. Sclerotic dentin forms due to the complete or partial obliteration of the dentinal tubules by intratubular deposition of crystals with tube-like or rod-like morphology and peritubular dentin. This dentin layer acts as a protective layer for the pulp, protecting it from external stimuli [18,19]. In this context, self-adhesive resins were introduced to promote an adhesive bond to dentin covered by a smear layer without pre-treatment [20]. Several studies have demonstrated that these cements exhibit limited interaction with the dentinal surface, failing to form a resin-dentin hybrid layer [21]. Instead, they primarily modify the smear layer at the cement-dentin interface [22].

Camargo et al. proposed smear layer removal to address this issue [23]. However, other researchers [24] suggested that the sclerotic dentin is the natural protective mechanism that should be preserved and clinicians should prohibit the cavity preparation, especially using the medium-grit diamond bur [25,26]. At present, no standard procedure exists, which improves the adhesive efficacy of sclerotic dentin.

In the present study, 37% phosphoric acid and 10% citric acid were used as etching agents and then surface treatment was done using three different bonding agents. Concurrently, Wang et al. demonstrated that pre-treatment with 37% phosphoric acid enhanced micro-tensile bond strength to both normal and sclerotic dentin [27]. Phosphoric acid pre-conditioning enhances the bond strength at the dentin-resin interface by removing peritubular dentin.

The potential application of citric acid as an etching agent in the smear layer removal was studied by Yao et al. They observed that the micro-hardness and the modulus of elasticity of the remineralised dentin were comparable to that of sound dentin [28]. There was a reduction in the microleakage with improved strong bonding in the remineralised interface thereby presenting a new approach for clinical implication. Similarly, the present study proved that citric acid had comparable efficacy to phosphoric acid as a surface pre-treatment agent.

Previous studies have demonstrated improved bonding strength to both normal and sclerotic dentin following EDTA pre-treatment [29]. However, Tori et al. reported no significant increase in bond strength when EDTA conditioning was used instead of phosphoric acid on bovine dentin before applying Single Bond [30]. The findings of the present study support the evidence that EDTA pre-treatment, regardless of the subsequent etching protocol, significantly increased the micro-tensile bond strength across all bonding agent groups employed. 

However, this study did not assess the parameters for clinical efficacy. Cohesive failure is characterised by a fracture within the tooth substrate or resin composite itself. This is different from adhesive failure, where the bond between the adhesive material and either the tooth or the restorative material separates without dentin fracture. Apart from this, mixed failure mode that includes both cohesive and adhesive failure needs to be studied to assess resin failure if present.

Another limitation of the present study is the lack of scanning electron microscopy (SEM) analysis to assess the efficacy of pre-treatment measures on the dentinal tubules at an ultramorphological level. It provides valuable insights regarding the changes in the dentinal smear layer, surfaces and sub-surfaces of dentin, and the formation of the resin layer. SEM assesses the relationship between the tooth and resin layer and the fracture mode.

## Conclusions

Restoration of NCCLs presents a significant challenge due to the presence of sclerotic dentin. This study investigated the influence of dentin pre-treatment and bonding agent selection on micro-tensile bond strength in NCCLs. The findings of the present study demonstrate that pre-treatment with 37% phosphoric acid resulted in significantly higher bond strength compared to 10% citric acid, irrespective of the dentin bonding agent employed. Additionally, Ivoclar Tetric N Bond® exhibited superior bond strength compared to other tested bonding agents.
